# Design of a Remote Multiparametric Tool to Assess Mental Well-Being and Distress in Young People (mHealth Methods in Mental Health Research Project): Protocol for an Observational Study

**DOI:** 10.2196/51298

**Published:** 2024-03-29

**Authors:** Thais Castro Ribeiro, Esther García Pagès, Laura Ballester, Gemma Vilagut, Helena García Mieres, Víctor Suárez Aragonès, Franco Amigo, Raquel Bailón, Philippe Mortier, Víctor Pérez Sola, Antoni Serrano-Blanco, Jordi Alonso, Jordi Aguiló

**Affiliations:** 1 CIBER de Bioingeniería, Biomateriales y Nanomedicina (CIBER-BBN) Instituto de Salud Carlos III Madrid Spain; 2 Departament of Microelectronics and Electronic Systems Autonomous University of Barcelona Bellaterra Spain; 3 CIBER de Epidemiología y Salud Pública (CIBERESP) Instituto de Salud Carlos III Madrid Spain; 4 Health Services Research Group Hospital del Mar Research Institute Barcelona Spain; 5 Department of Clinical Psychology and Psychobiology University of Barcelona Barcelona Spain; 6 Aragón Institute of Engineering Research (I3A) University of Zaragoza Zaragoza Spain; 7 CIBER en Salud Mental (CIBERSAM) Instituto de Salud Carlos III Madrid Spain; 8 Institute of Neuropsychiatry and Addictions (INAD) Parc de Salut Mar (PSMAR) Barcelona Spain; 9 Neurosciences Research Group Hospital del Mar Research Institute Barcelona Spain; 10 Department of Medicine and Life Sciences Universitat Pompeu Fabra Barcelona Spain; 11 Institut de Recerca Sant Joan de Déu Parc Sanitari Sant Joan de Déu Barcelona Spain

**Keywords:** mental health, mental well-being, mobile health, mHealth, remote monitoring, physiological variables, experimental protocol, depression, anxiety

## Abstract

**Background:**

Mental health conditions have become a substantial cause of disability worldwide, resulting in economic burden and strain on the public health system. Incorporating cognitive and physiological biomarkers using noninvasive sensors combined with self-reported questionnaires can provide a more accurate characterization of the individual’s well-being. Biomarkers such as heart rate variability or those extracted from the electrodermal activity signal are commonly considered as indices of autonomic nervous system functioning, providing objective indicators of stress response. A model combining a set of these biomarkers can constitute a comprehensive tool to remotely assess mental well-being and distress.

**Objective:**

This study aims to design and validate a remote multiparametric tool, including physiological and cognitive variables, to objectively assess mental well-being and distress.

**Methods:**

This ongoing observational study pursues to enroll 60 young participants (aged 18-34 years) in 3 groups, including participants with high mental well-being, participants with mild to moderate psychological distress, and participants diagnosed with depression or anxiety disorder. The inclusion and exclusion criteria are being evaluated through a web-based questionnaire, and for those with a mental health condition, the criteria are identified by psychologists. The assessment consists of collecting mental health self-reported measures and physiological data during a baseline state, the Stroop Color and Word Test as a stress-inducing stage, and a final recovery period. Several variables related to heart rate variability, pulse arrival time, breathing, electrodermal activity, and peripheral temperature are collected using medical and wearable devices. A second assessment is carried out after 1 month. The assessment tool will be developed using self-reported questionnaires assessing well-being (short version of Warwick-Edinburgh Mental Well-being Scale), anxiety (Generalized Anxiety Disorder-7), and depression (Patient Health Questionnaire-9) as the reference. We will perform correlation and principal component analysis to reduce the number of variables, followed by the calculation of multiple regression models. Test-retest reliability, known-group validity, and predictive validity will be assessed.

**Results:**

Participant recruitment is being carried out on a university campus and in mental health services. Recruitment commenced in October 2022 and is expected to be completed by June 2024. As of July 2023, we have recruited 41 participants. Most participants correspond to the group with mild to moderate psychological distress (n=20, 49%), followed by the high mental well-being group (n=13, 32%) and those diagnosed with a mental health condition (n=8, 20%). Data preprocessing is currently ongoing, and publication of the first results is expected by September 2024.

**Conclusions:**

This study will establish an initial framework for a comprehensive mental health assessment tool, taking measurements from sophisticated devices, with the goal of progressing toward a remotely accessible and objectively measured approach that maintains an acceptable level of accuracy in clinical practice and epidemiological studies.

**Trial Registration:**

OSF Registries N3GCH; https://doi.org/10.17605/OSF.IO/N3GCH

**International Registered Report Identifier (IRRID):**

DERR1-10.2196/51298

## Introduction

### Background

Mental health conditions are one of the leading causes of disability worldwide and are estimated to reduce life expectancy by 10 years [1]. For instance, depressive disorders were considered the eighth cause of disability in Spain in 2000, rising to fifth in 2019 [2]. Depression, anxiety, and stress-related disorders impose a major economic impact and burden on the public health system. To date, the prevalence of depression in the Spanish population is close to 5%, and the annual cost is estimated at €6145 (US $6648) million [3].

Young people and university students are populations of particular interest. Approximately 75% of mental health conditions have an early onset before the age of 24 years, and several risk factors (including genetic, early life adversity, family, community, and environmental factors) are involved in the development and course of these conditions [4]. Moreover, the recent COVID-19 pandemic has aggravated this situation [5]. Several systematic reviews and meta-analyses have indicated a high prevalence of mental health conditions among young people, with a pooled prevalence for depression of 31% to 33.6%, anxiety of 28% to 39%, sleep problems of 40%, and suicidal ideation of 12.3% [6-8], in line with the results of longitudinal studies suggesting a possible worsening of mental health in this population in recent years [9]. There is a need for early identification and prevention of mental health conditions, which includes the design and implementation of mental health promotion activities that lead to an increase in emotional well-being [10].

In recent decades, interest in mental health research has been steadily increasing, recognizing it a crucial aspect of overall health, rather than simply the absence of related conditions [11]. According to the World Health Organization [12], mental health is characterized by individuals’ ability to effectively manage typical stressful situations, develop their potential and skills, and contribute productively to both themselves and the community. This comprises an adequate stress response and recovery as well as maintaining cognitive abilities such as attentional level and proper time of response. Stress reactivity is this capacity to respond to a stressor. It is a disposition that underlies individual differences in response to stressors and is assumed to be a vulnerability factor for the development of mental health conditions [13]. In this context, monitoring physiological response during a stress-inducing task could yield different reactivity patterns, offering valuable insights to differentiate between mental well-being and distress.

In clinical practice, self-reported questionnaires are commonly used to assess the severity of mental health symptoms, quality of life, and mental well-being. Nevertheless, several studies have reported limitations of these tests related to memory biases and distortions in retrospective recall [14,15]. To expand such assessments, including physiological biomarkers information will improve the characterization of endophenotypes (research domain criteria) [16]. Owing to technological advances, small sensors can measure physiological data for behavioral health, interventions, and outcomes (digital phenotyping) [17,18]. Given the significance of stress reactions as complex phenomena encompassing psychological, cognitive, and physiological reactions involving the autonomic nervous system (ANS) and the neuroendocrine system, which, in turn, can affect other bodily systems, exploring these dynamics could enhance our comprehension of mental distress. Hence, physiological data monitoring including a stress-eliciting task may have an important role in early detection and intervention in mental health care. Heart rate variability (HRV), pulse arrival time (PAT), breathing parameters, electrodermal activity (EDA), and skin temperature (ST) are physiological variables broadly used to study the stress response and gather information about ANS functioning [19-24].

To progress in this field, the use of wearables in mental health research shows promise, offering increased accuracy in data collection and reduced participant burden. Wearable devices allow researchers to passively monitor individuals in real time and gather data outside of traditional laboratory settings, that is, along with everyday life situations, providing a more holistic understanding of mental health status [25,26]. Currently, many studies on stress detection are conducted in controlled environments because accuracy decreases when conducted in real-time environments [27]. In addition, different instruments to measure perceived stress are used, which hinders the comparability of results [28], or a small number of signals are usually collected [29-31]. However, previous studies have shown optimistic results for further advancement in the field for the objective assessment of mental health status and stress. A study analyzing data from 510 participants wearing a Fitbit device during a 2-year follow-up [32] showed a correlation between decreased resting heart rate variation during the day and the severity of depression, whereas the mean heart rate at night was higher in participants with more severe depressive symptoms. In line with these results, a decreased autonomic reactivity measured through dynamic changes in photoplethysmography (PPG) waveform morphology was associated with a higher degree of depression in the study by Kontaxis et al [33]. Sano et al [30] conducted an observational study among university students using wearable sensors that collected EDA and ST, and using psychometric questionnaires as reference, they found an accuracy of 78% and 87% to classify into high or low stress groups and high or low mental health groups, respectively. Similarly, Sano et al [34] found an accuracy of 90% in classifying stress and mental health groups. From a literature review [27], it was observed that heart rate and EDA are the most regularly used sensory signals, offering the most promising results and high accuracy for detecting stress.

Effective prevention interventions require strategies to identify early risk groups according to risk factors through the development of predictive models. In addition, from a mental health promotion perspective, effectively assessing mental well-being would help identify the right time to intervene, evaluate the efficacy of the therapy applied, empower the citizens, offer stress-reducing programs, and prevent negative consequences. Here, we present the development and evaluation of a novel multiparametric tool to improve mental health assessments and to facilitate the evaluation of risk and protective factors as well as the effectiveness of promotion and prevention interventions.

### Objectives

This study aims to design and validate a remote multiparametric tool, including several physiological and cognitive variables, to objectively assess mental well-being as well as mental distress (ie, symptoms of depression and anxiety) among young people for epidemiologic and clinical studies.

The specific objectives of this study are (1) to develop an assessment tool for mental well-being and distress based on the most relevant physiological and cognitive variables; (2) to validate the assessment tool using self-reported measures and evaluate the tool reliability and accuracy; and (3) to develop and establish a protocol to automate the measurement process, ensuring that it can be reproduced in large populations.

## Methods

### Study Design and Setting

This is a multicenter observational study of the mHealth Methods in Mental Health Research (M&M) project, currently ongoing, being conducted by the Autonomous University of Barcelona (UAB) and Parc de Salut Mar (PSMAR).

Three different mental health states will be studied: (1) high mental well-being, (2) presenting mild to moderate psychological distress, and (3) depressive or anxiety disorder (diagnosed by a mental health professional). For the high mental well-being and the mild to moderate psychological distress groups, a web-based mental health questionnaire is being distributed among UAB students for screening and analyzed to determine the participant’s eligibility. Participants who meet the selection criteria are consecutively included. For the mental health condition group, the patients are being referred from the Institute of Neuropsychiatry and Addictions-PSMAR, the Hospital Sant Joan de Déu, and the Psychology and Speech Therapy Service of the UAB. The assessments are planned at 2 time points and are being conducted at the site of recruitment (UAB, Institute of Neuropsychiatry and Addictions-PSMAR, or Hospital Sant Joan de Déu). The second assessment takes place after 1 month of the first assessment.

### Participants and Eligibility Criteria

The 3 abovementioned participant groups are being recruited according to the inclusion and exclusion criteria described in detail in Table 1. To ensure a homogeneous sample in terms of age, participants aged between 18 and 34 years are being recruited in all 3 groups.

**Table 1:** Inclusion and exclusion criteria for the 3 groups of the participants being recruited in the multicenter observational study (mHealth Methods in Mental Health Research project).

Groups	Inclusion criteria	Exclusion criteria	Site of recruitment
High mental well-being	No history of emotional distress for at least a year PHQ-4^a^ score: <3 and SWEMWBS^b^ score: ≥30	Cognitive impairment or damage, including presence or history of head trauma, dementia, or intellectual disability (IQ ≤80) History of schizophrenia or other psychotic spectrum disorders Problems understanding Spanish or Catalan	University campus (UAB^c^)
Mild to moderate psychological distress	Recent history of mental health issues PHQ-4 score: 3-8 or SWEMWBS score: 20-29	Cognitive impairment or damage, including presence or history of head trauma, dementia, or intellectual disability (IQ ≤80) History of schizophrenia or other psychotic spectrum disorders Problems understanding Spanish or Catalan	University campus (UAB)
Mental health condition	Diagnosed with current depression or anxiety by a mental health professional	Cognitive impairment or damage, including presence or history of head trauma, dementia, or intellectual disability (IQ ≤80) History of schizophrenia or other psychotic spectrum disorders Problems understanding Spanish or Catalan The symptomatology has an organic origin or is owing to the physiological effects of a substance Presence of acute suicidal ideation Being medicated	Mental health services (INAD-PSMAR^d^ or HSJD^e^)

^a^PHQ-4: Patient Health Questionnaire-4.

^b^SWEMWBS: short version of Warwick-Edinburgh Mental Well-being Scale.

^c^UAB: Autonomous University of Barcelona.

^d^INAD-PSMAR: Institute of Neuropsychiatry and Addictions-Parc de Salut Mar.

^e^HSJD: Hospital Sant Joan de Déu.

For the high mental well-being and mild to moderate psychological distress groups, inclusion criteria are assessed for eligibility through a web-based questionnaire that contains questions about mental health history (eg, “Have you ever experienced any mental health issue?”). The Patient Health Questionnaire (PHQ; PHQ-4) [35] is used to screen for anxiety and depression symptoms, and the short version of Warwick-Edinburgh Mental Well-being Scale (SWEMWBS) [36,37] is used to evaluate mental well-being. The cutoff points to be considered as high mental well-being are based on data from a representative sample of young adults of Catalonia from the Catalonia Health Survey conducted in 2016 [38], in which a median score of 30 points in SWEMWBS was found. Individuals with SWEMWBS well-being score ≥30 and PHQ-4 <3 points are classified in the high well-being group. Individuals with SWEMWBS score between 20 and 29 points or a PHQ-4 score between 3 and 8 are classified into the mild to moderate psychological distress group.

### Recruitment

The primary recruitment pathway for nonpatients is the dissemination of the study through institutional mail or social media and the placement of posters in public areas of UAB. The information includes a link or QR code to answer a web-based questionnaire. To facilitate the recruitment of the mild to moderate psychological distress group, the Psychology and Speech Therapy Service of the UAB is collaborating by inviting students who attended the service to participate in this study. In both cases, once the responsible researcher confirms the eligibility criteria, the participant is contacted to schedule the first assessment.

For the mental health condition group, the patients who meet the criteria are identified at the consultation with the psychologist or psychiatrist, who briefly informs them about the study and suggests participation. The research assistant contacts the interested patients by phone and makes an appointment for the first assessment. Written informed consent is provided by all participants before starting the first assessment interview.

### Study Procedure

**Figure 1:**
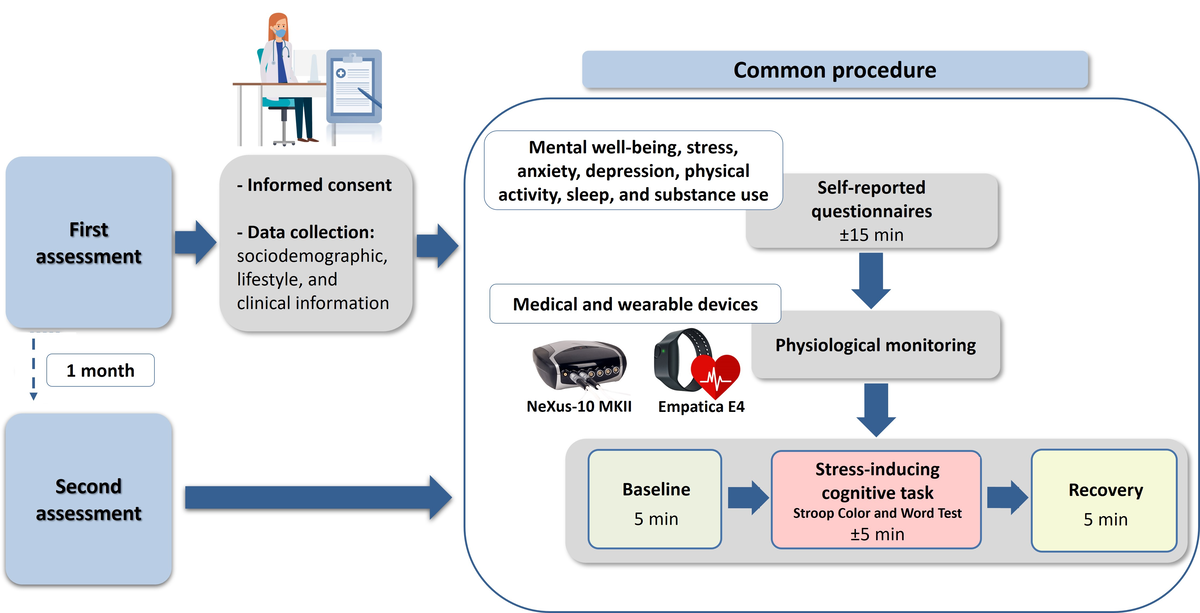
The study experimental procedure outline for the first and second (conducted after 1 month) assessments includes recruitment, obtaining informed consent, and data collection. A research assistant performs the same procedure for both assessments in this observational study (mHealth Methods in Mental Health Research [M&M] project).

All participants who agree to participate are asked to abstain from tobacco, alcohol, caffeine, or any other beverage or stimulating substance for 2 hours before the study. Figure 1 shows the complete schematic of the experimental procedure.

At the first assessment, the participants are fully informed about the study procedure and are requested to sign the informed consent form. This visit includes an ad hoc interview conducted by a qualified examiner to collect individuals’ sociodemographic, modifiable lifestyle factors, health-related variables, and clinical data through the management software Qualtrics (Silver Lake). Subsequently, a psychological assessment is carried out. The participants respond to 7 self-reported questionnaires using the same software. These questionnaires aim to estimate the current mental well-being, stress perception, symptoms of anxiety and depression, physical activity, sleep quality, and substance use. All these measures are described in the *Study Variables* section.

The physiological assessment consists of recording different stress-related physiological signals using (1) the medical-graded device NeXus-10 MKII (Mind Media BV) and (2) the wearable E4 Empatica wristband (Empatica Inc). PPG, EDA, and ST will be the physiological signals recorded simultaneously by both devices. The electrocardiogram (ECG) and respiration can only be measured using a medical-graded device. This device is used to obtain a more accurate measure for preliminary analysis and, thereafter, validate the predictive model with the wearable device.

The wristband is placed on the nondominant wrist and the PPG (middle finger), EDA (middle phalanges of the second and fourth digits), and ST (fingertip of the fifth finger) sensors are placed on the nondominant hand to avoid excessive movement artifacts. An adjustable elastic band is placed over the abdomen to measure the respiration signal. For lead 1 of the ECG signal, electrodes are positioned below the right collarbone and below the left rib cage, whereas for lead 2, electrodes are positioned on the fifth intercostal space along the midaxillary line on the left side and symmetrically on the right side. The reference electrode is placed on the left collarbone.

This part of the procedure lasts approximately 15 minutes and is divided into three different stages: (1) baseline (green block in Figure 1): participant in a resting state, sitting comfortably with eyes open; (2) cognitive task (red block in Figure 1): corresponds to the stress-inducing stage, when the individual is submitted to a cognitive task, the Stroop Test [39]; and (3) recovery (yellow block in Figure 1): when the individual’s physiological responses are expected to return to the baseline levels. All physiological signals and variables of interest will be detailed in the *Physiological Variables* section.

A second assessment is then scheduled 1 month apart and includes the same psychological and physiological assessments. This follow-up session is intended to allow test-retest reliability and account for random errors that could occur in a single session.

### Study Variables

#### Outcome Measures

The following outcome measures are used:

1. Depression: It is evaluated using the PHQ-9 [40,41]. It is a Likert-type scale used to screen the severity of depressive symptoms according to the *Diagnostic and Statistical Manual of Mental Disorders, Fourth Edition*. All 9 items are rated from 0 (not at all) to 3 (nearly every day). Total scores can range from 0 to 27, with higher scores indicating more severe depression. Furthermore, 5, 10, 15, and 20 represent the cutoff points for mild, moderate, moderately severe, and severe depression, respectively [42].
2. Anxiety: The Generalized Anxiety Disorder-7 [43,44] is an instrument for screening the presence of symptoms of anxiety as listed in the *Diagnostic and Statistical Manual of Mental Disorders, Fourth Edition*. It is a 1-dimensional scale with scores for all 7 items ranging from 0 (not at all) to 3 (nearly every day). The total score was categorized into 4 severity groups according to the original authors: minimal (0-4), mild (5-9), moderate (10-14), and severe (>15).
3. Mental well-being: The SWEMWBS [36,37] is an instrument used to assess mental well-being. This unidimensional scale comprises 7 items ranging from 1 (none of the time) to 5 (all of the time). The higher the total score, the greater the perception of well-being.

#### Covariates

Sociodemographic variables include age, gender, nationality, marital status, living status, educational level, and occupation.

Current physical and mental health conditions, previous mental health treatments, medication, and suicidal thoughts and behaviors are evaluated through items in the ad hoc interview. Perceived stress is evaluated using the 10-item Perceived Stress Scale [45,46]. It is a 5-point Likert scale with questions about the frequency of feelings and thoughts during the last month, with each item ranging from 0 (never) to 4 (very often). Higher scores indicate higher levels of perceived stress. To assess substance use problems, the CAGE-Adapted to Include Drugs [47] scale is used. It is an adaptation of the original CAGE questionnaire [48] for conjointly screening for alcohol and drug problems based on lifetime. The scale contains 4 yes or no questions, and a higher score indicates substance use problems.

Modifiable lifestyle variables are assessed using an ad hoc interview, including coffee consumption, cigarette smoking, alcohol and drug consumption, and BMI. Physical activity is measured using the short form of the International Physical Activity Questionnaire [49,50]. This questionnaire comprises 7 open-ended questions about individuals’ 7-day recall of physical activity. According to the total energy expenditure in metabolic equivalent of task (ie, 1 metabolic equivalent of task is the energy cost of sitting quietly) in minutes per week, the physical activity level is determined as low or inactive, moderate, or high.

Sleep is evaluated using the Medical Outcomes Study Sleep Scale [51,52]. This questionnaire contains 12 items about a 4-week recall, divided into 8 subscales (sleep adequacy, optimal sleep, quantity of sleep, awakening shortness of breath or with headache, snoring, sleep disturbance, somnolence, and global index of sleep interference). In general, higher scores indicate greater sleep problems. The quantity of sleep score is the patient-reported number of hours of sleep per night, and optimal sleep is scored as 1 (7 or 8 h of sleep per night) or 0 (any different response).

The comfort level is evaluated by asking participants if they are currently experiencing higher stress than usual and identifying its potential causes.

#### Stress-Inducing Cognitive Task

The Spanish version of the standard Stroop Color and Word Test (SCWT) is applied (originally [53] and Spanish version [39]) as a cognitive stress-inducing task. This test is extensively used to assess cognitive inhibition and processing speed. Furthermore, it has also been shown to be a reliable method to induce mental stress in experimental settings [54]. The individuals are required to read the 3-color cards as fast as possible in a fixed time of 45 seconds each. The stimuli presented on the first 2 cards are congruent, that is, read names of colors or name different colors. In contrast, the last card represents the incongruent stimuli, that is, name the color of the ink instead of reading the color.

Three direct scores are derived by tallying the correct responses for each condition: (1) *W* (word) represents the number of colors read on the first card (where colors are written in black ink), (2) *C* (color) represents the number of elements identified on the card of colors (where name colors are represented with strings of XXXXs), and (3) *CW* (color word) represents the number of items correctly identified on the third card (where colors are printed in an ink that does not correspond to the color name, requiring participants to say the colors of the ink). Two other scores will be calculated from these: (1) predicted *CW* (*PCW*): (*W* × *C* / *W* + *C*) and (2) interference: (*CW* − *PCW*). A higher score indicates a greater ability to inhibit interference.

Direct scores are then converted to *T* scores, with a preset mean of 50 and SD of 10, so that they can be more easily compared in similar age ranges (in this study, young adults aged between 18 and 44 years). The limits considered normal are between 35 and 65 *T* points in any of the scores (for details, refer the study by Golden [39]).

#### Physiological Variables

##### Overview

We are following a methodology already used and validated as stress assessment for healthy students and principal caregivers [20,55]; the electrophysiological raw signals recorded with NeXus-10 MKII (ie, ECG, PPG, EDA, respiration, and ST) are analyzed using BioSigBrowser [56] in MATLAB software (The MathWorks Inc); and several groups of variables are extracted, as described in Table 2. A literature review was conducted to select the most relevant variables for assessing stress response and mental health. A summary of the findings can be found in Multimedia Appendix 1 [19-24,57-65].

**Table 2:** Description of the variables that will be extracted from raw electrophysiological signals monitored with the medical device NeXus-10 MKII.

Group of variables	Electrophysiological signal	Physiological variables	Description
HRV^a^ or PRV^b^	ECG^c^ or PPG^d^	HR^e,f^ (bpm)^g^	Mean HR
HRV or PRV	ECG or PPG	IBI^f^ (ms)^h^	Mean IBI
HRV or PRV	ECG or PPG	pNN50^f^ (%)	Percentage of adjacent normal beats intervals differing from each other by >50 ms
HRV or PRV	ECG or PPG	SDNN^f^ (ms)	SD of normal beats intervals
HRV or PRV	ECG or PPG	RMSSD^f^ (ms)	Root mean square of successive differences between normal beats
HRV or PRV	ECG or PPG	SDSD^f^ (ms)	SD of differences between adjacent R-R peaks intervals
HRV or PRV	ECG or PPG	VLF^i^ (s^−2^)	Absolute power of the VLF band (0.003-0.04 Hz)
HRV or PRV	ECG or PPG	LF^f,j^ (s^−2^)	Absolute power of the LF band (0.04-0.15 Hz)
HRV or PRV	ECG or PPG	HF^f^ (s^−2^)^k^	Absolute power of the HF band (0.15-0.4 Hz)
HRV or PRV	ECG or PPG	LF/HF ratio^f^	Ratio of LF to HF power
HRV or PRV	ECG or PPG	HFn^f^ (nu)	Relative power of the HF band normalized
PAT^l^	ECG and PPG	PAT (ms)	Mean PAT, the time between the beat detected by ECG and the pulse by PPG
PAT	ECG and PPG	stdPAT (ms)	SD of PAT
Breathing	Respiration	RR^m^ (Hz)	Mean RR
Breathing	Respiration	Pk (%)	Peak of respiratory power spectra
EDA^n^	EDA	Tonic^f^ (µS)	Average value of the tonic component, that is, slowly changing skin conductance level, also known as SCL^o^
EDA	EDA	Phasic^f^ (µS)	Average value of the phasic component, that is, fast-changing responses typically associated with short-term events, also known as SCR^p^
EDA	EDA	aucPhasic^f^ (µS·s)	Area under the curve of the phasic component, which is related to SCR
EDA	EDA	EDASymp^f^ (µS)	Electrodermal response in the power spectrum (0.045-0.25 Hz)
Peripheral temperature	ST^q^	TFinger^f^ (°C)	Mean finger temperature
Peripheral temperature	ST	TGradient^f^ (°C)	Mean gradient of finger temperature
Peripheral temperature	ST	TPower^f^ (°C^2^)	Mean power of finger temperature

^a^HRV: heart rate variability.

^b^PRV: pulse rate variability.

^c^ECG: electrocardiogram.

^d^PPG: photoplethysmography.

^e^HR: heart rate.

^f^These will also be extracted from the recordings of the Empatica E4 wearable device.

^g^bpm: beats per minute.

^h^IBI: interbeat interval.

^i^VLF: very low frequency

^j^LF: low frequency.

^k^HF: high frequency.

^l^PAT: pulse arrival time.

^m^RR: respiratory rate.

^n^EDA: electrodermal activity.

^o^SCL: skin conductance level.

^p^SCR: skin conductance response.

^q^ST: skin temperature.

The raw signals recorded with the Empatica E4 wearable device (ie, EDA, PPG, and ST) will be also analyzed in MATLAB (The MathWorks Inc) using a similar procedure, given the different format files. The variables intended to be explored in this case are indicated in the footnotes in Table 2. Furthermore, the stress reactivity, that is, the difference between the stress-inducing cognitive task stage and the baseline stage (stress−baseline), and the stress recovery, that is, the difference between the stress and the posterior recovery stage (stress−recovery), will also be computed for each variable to determine the most relevant set of variables to be considered to design the final model.

##### Physiological Data Processing

For processing the ECG signal, beat detection is performed through a discrete wavelet transform [66]. Afterward, the existence of ectopic beats or false QRS detections will be verified and corrected using the algorithm reported by Mateo and Laguna [67] before the computation of the interbeat interval series. Segments of up to 3 interpolated or corrected beats are accepted and assumed to be normal. Following this, the HRV parameters are calculated by a time-domain analysis and a frequency-domain analysis by Fourier transform of the heart rate signal.

PPG signal is preprocessed using a low-pass finite impulse response (FIR) filter with a cutoff frequency of 35 Hz (order 50) and then a high-pass FIR filter with a cutoff frequency of 0.3 Hz (order 5000). PPG artifacts are suppressed using a Hjorth parameter–based PPG artifact detector described by Gil et al [68]. Pulses are detected from the PPG signal on those time slots without artifacts using an algorithm based on the study by Lázaro et al [69]. The same ECG parameters are also extracted in PPG, in this case, referred to as pulse rate variability. Subsequently, the mean time difference between the R peak in the ECG signal and the point of 50% increase, corresponding to the pulse detected on the finger by the PPG signal, is considered as the PAT, and its SD (SD of PAT) is also calculated.

The respiration wave is filtered with an FIR passband filter with cutoff frequencies of 0.03 and 0.9 Hz. The respiratory rate is estimated as the frequency to which the maximum peak of the power density spectrum corresponds, estimated using a fast Fourier transform [70]. When the peak is >65%, then respiratory rate is considered valid.

The EDA signal is visually inspected to remove motion artifacts and linearly interpolated. First, a time-domain analysis is performed using a convex optimization model, called cvxEDA [57], to calculate the tonic and phasic components. The second procedure is a frequency-domain analysis, proposed to assess sympathetic tone through a parameter named EDASymp, described in the study by Posada-Quintero et al [71].

Finally, for the ST signal, a visual inspection is carried out to look for possible large artifacts. These segments are discarded before proceeding with the calculation of the parameters.

### Statistical Analysis Plan

An initial descriptive analysis will be conducted for all study variables, for the overall sample and stratified by study group. The quantitative variables will be summarized, assuming normal distribution (Shapiro-Wilk normality test), using the mean and SD. The qualitative variables will be summarized using the relative and absolute frequencies. Physiological variables that present a skewed distribution will be logarithmically transformed.

A parametric test (Pearson correlation) or nonparametric test (Spearman correlation) will be, accordingly, applied as a descriptive measure of the association between quantitative variables.

To develop a useful tool for assessing mental distress and well-being, the initial step of the statistical analysis plan will involve variables reduction using two methods: (1) a correlation analysis to prioritize the most relevant variables for the prediction of the primary outcome measures and (2) a principal component analysis to find the directions of maximum variance in the data and reduce collinearity. Subsequently, the generated components that account for at least 85% (51/60) of the sample’s variability will be included. From these components, 2 separate analyses will be conducted. First, a generalized linear model will be fitted to predict the values of each primary outcome measure. Second, a model will be developed to differentiate the 3 groups identified in the study, each representing a different level of mental health. The models will be fitted using standardized variables and performing k-fold cross-validation to quantify the model’s performance using *R*^2^ for the linear regression model and area under the curve for the classification models. Known-group validity will be assessed by comparing the mean scores of the tool among the preestablished groups at baseline: diagnosed with current depression or anxiety, symptoms of mental distress, and high mental well-being. The predefined hypothesis that higher model scores are predicted for individuals with higher well-being will be evaluated using the Jonckheere-Terpstra test, and Cohen effect sizes will be computed for each category as compared with the lowest category (mental health condition), considering small (0.2), moderate (0.5), and large (0.8) effect sizes [72].

The model test-retest reliability will be assessed with a 2-way random effect intraclass correlation coefficient (ICC), taking repeated evaluations of the same *unchanged* individuals, to assess the extent to which measures remain stable. A *change* will be considered a clinically relevant change in the outcome scores, ie, ≥4 points for the Generalized Anxiety Disorder-7, ≥5 points for PHQ-9, or ≥3 points for SWEMWBS) [73-75].

The significance level will be set at α=.05. Statistical analysis will be performed using SAS (version 9.4; SAS Institute).

### Sample Size

As a proof-of-concept study, we plan to include 20 individuals per group (a total of 60 individuals×2 evaluations). For the assessment of known-groups validity of the developed tool, with this sample size of 20 individuals per group, and a type I error rate α=.05 on a 1-sided *t* test, we will have power of 0.80 to detect a difference between 2 groups corresponding to an effect size of 0.8. For a moderate effect size of 0.5, the power will decrease to 0.47 [17].

Concerning the assessment of test-retest reliability of the tool, assuming a 15% (9/60) loss in follow-up or nonstable participants from baseline participants, an ICC of 0.6 under the null hypothesis, and a type I error rate of α=.05, a sample size of 51 participants with 2 observations per participant achieves a power of 0.90 to detect a hypothetical ICC value of 0.8 under the alternative hypothesis [17].

### Ethical Considerations

This study protocol was approved by the research ethics committees of both institutions (2021/10163 for PSMAR and 5912 for UAB). This study is in line with the principles established by national and international regulations, including the Declaration of Helsinki and the Code of Ethics. Ethics approval has been obtained in Barcelona from the independent PSMAR Clinical Research Ethics Committee and the Research Ethics Committee of the UAB. Informed consent is requested from all participants before their inclusion in the study. Participants are explained that they can withdraw from the study at any time without giving a reason and that they can request to delete all the data collected from them.

All personal data will be handled following Regulation (European Union) 2016/679 of the European Parliament and the Council on the protection of natural persons concerning the processing of personal data and on the free movement of such data and the National Organic Law 3/2018, of December 5, on Personal Data Protection and the Guarantee of Digital Rights. Physiological and psychometric data will be pseudoanonymized to guarantee privacy in data analysis and will be stored in a research database following the General Data Protection Regulation of the European Union.

Participants receive a €10 (US $11) gift card for enrolling in the study, and participants receive another gift card of the same value if they complete the second assessment.

## Results

The project was granted in February 2022 and the approval from ethical committees was obtained between April and May 2022. Participant recruitment started in October 2022 and is expected to continue through June 2024. Different recruitment strategies are implemented, including advertising campaigns and invitation letters at the university and recruitment of patients by psychologists.

As of July 2023, a total of 41 participants completed the first and second assessments. The sample corresponds mainly to the group with mild to moderate psychological distress (n=20, 49%), followed by the group with high mental well-being (n=13, 32%) and, finally, those diagnosed with an anxiety disorder (n=8, 20%). At this point, preprocessing and quality checks of the data are ongoing, and the statistical analysis will subsequently begin. The first results are expected to be published in September 2024.

## Discussion

### Overview

There is a growing interest in remotely assessing mental health through changes in the ANS functioning and its association with mental health and well-being [26]. There is significant evidence to support the notion that changes in the ANS can be inferred from changes in physiological variables, such as HRV, EDA response, and peripheral temperature [58,76,77]. However, there is a challenge in designing a robust predictive model that allows this assessment to be easy and objective to be systematically used in epidemiology and clinical settings. To fill this gap, it is important to carefully measure electrophysiological signals considering the updated standards of measurement (eg, [78]), following a structure of 3-time point experimental design: considering a basal condition, during a stress-inducing task to investigate the stress reactivity, and a recovery stage. This study is applying a similar methodology used in previous research that reached good reliability in its predictive models [20,79]. However, in this novel approach, individuals with a clinically diagnosed mental health condition are being enrolled, which may allow for a better distinction between different profiles of mental health through the stress reactivity pattern and cognitive performance.

The SCWT is a well-known neuropsychological test reported as a reliable moderate mental stressor, provoking significant physiological changes such as reduced HRV and increased EDA and blood pressure [59,80,81]. Furthermore, it is a useful tool for evaluating cognitive processes and has the advantage of not generating a significant learning effect [82]. Cognitive inhibition is compromised in multiple mental health conditions; for example, individuals with anxiety disorders tend to have longer reaction times and higher error rates on the SCWT, particularly in the incongruent condition, which suggests difficulties with selective attention and response inhibition [83]. Thus, although the impaired performance of the task may be indicative of an underlying neurological disorder, a good result performance may add another layer of confidence in evaluating mental well-being.

There is a lot of research committed to investigating biomarkers and stress reactivity in patients diagnosed with depression and anxiety disorders [15,84]. The evidence supports differences in physiological behavior in patients with depression and anxiety compared with healthy individuals. Considering this, the decision to include 3 groups with different mental health states will facilitate the detection of patterns that could discriminate them more accurately. The age range selected is justified by the typical early age of onset of mental health conditions and the need for assertive responses to prevent a poor prognosis. Moreover, the recruitment is planned to be carried out in part at a university, considering that university students have been reported as a susceptible population with a higher risk to manifest mental health symptoms [85], which enables us to recruit the mild to moderate psychological distress group effortlessly. To minimize interindividual variation in physiological measures and the influence of external factors, we propose 2 different sessions with the same participants, which will also increase the statistical power [78].

Mobile health (mHealth), which includes physical devices, sensors, software, and other technologies, has been proposed to improve clinical care as it enables data collection, symptom monitoring, and provision of interventions [29]. In this sense, it could be a valuable resource to reach both regular patients and those who do not receive adequate care [12]. Specifically, the use of wearables provides a new and unobtrusive way to monitor physiological biomarkers and gather continuous information about individuals’ daily lives and clinical symptoms for both clinical and research purposes. These devices with multiple embedded sensors can be useful in following up patients and remotely assessing their mental well-being through robust models that combine a set of relevant biomarkers [31].

Nevertheless, to ensure that autonomous nervous system biomarkers are effectively used for objectively measuring mental well-being in health services, it is necessary to undertake substantial work in identifying the most useful biomarkers and comprehending the possible obstacles and enablers of widespread adoption. To advance in this direction, this study intends to carry out a preliminary model validation by enrolling a preselected sample with different states of mental well-being in a laboratory-controlled condition; subsequently, we plan to explore its application in larger populations and in a real-life context.

To summarize, our study aims to design a comprehensive multiparametric model combining physiological and cognitive variables to assess the mental well-being among young people. The self-reported questionnaires currently used in clinical settings will be used as a reference to select the best model fit. This novel approach proposes shifting the paradigm to assessing mental well-being rather than measuring the severity of symptoms or a mental health condition. We believe that this change may allow health professionals to properly recommend prevention strategies and increase the possibility of intervening before the diagnosis of a mental health condition. To effectively develop a model that can be easily calculated by a wearable device, we first take a measurement of a standard medical device to ensure the best quality of physiological signal and then establish the final predictive tool.

### Conclusions

This study represents the primary phase in developing a comprehensive mental well-being assessment tool. Our goal is to progress toward remote measurements with acceptable accuracy, using sophisticated devices as a benchmark for comparison. Developing a robust predictive model will facilitate objective assessment that can be systematically used in epidemiological and clinical settings. Further research is required to explore the full potential of this technology in mental health research and clinical practice.

## Appendix

Multimedia Appendix 1 Summary of the literature review of the physiological variables assessed in the M&M mHealth Methods in Mental Health Research (M&M) Project

## Abbreviations

ANS: autonomic nervous system
ECG: electrocardiogram
EDA: electrodermal activity
FIR: finite impulse response
HRV: heart rate variability
ICC: intraclass correlation coefficient
M&M: mHealth Methods in Mental Health Research
mHealth: mobile health
PAT: pulse arrival time
PHQ: Patient Health Questionnaire
PPG: photoplethysmography
PSMAR: Parc de Salut Mar
SCWT: Stroop Color and Word Test
ST: skin temperature
SWEMWBS: short version of Warwick-Edinburgh Mental Well-being Scale
UAB: Autonomous University of Barcelona

## Data Availability

The data sets generated during this study will be deposited in the Open Science Framework public repository.
