# Supplementary material for: Design of a Remote Multiparametric Tool to Assess Mental Well-Being and Distress in Young People (mHealth Methods in Mental Health Research Project): Protocol for an Observational Study
Source: JMIR Res Protoc. 2024 Mar 29;13:e51298. doi: 10.2196/51298 (PMC11015365; doi:10.2196/51298)
Supplement: Multimedia Appendix 1 [file resprot_v13i1e51298_app1.docx]

# Multimedia Appendix:

## Summary of the literature review of the physiological variables assessed in the mHealth Methods in Mental Health Research (M&M) Project

This is a Multimedia Appendix to a full manuscript published in the JMIR Research Protocols. For full copyright and citation information see <http://doi.org/10.2196/51298>

The physiological variables included in the mHealth Methods in Mental Health Research Project are shown below:

1. **Heart rate variability (HRV),** which reflects the fluctuation in time intervals between adjacent heartbeats, is one of the most commonly used for this purpose [19]. It can be assessed by electrocardiogram (ECG) or photoplethysmography (PPG). A reduced heart rate variability has been associated with poor ANS functioning, negative cardiovascular outcomes and cognitive impairment. On the contrary, greater HRV has been linked with better performance, health and adaptability [58].
2. **Pulse arrival time (PAT)** has been extensively studied as a surrogate of blood pressure (BP), as it could estimate BP noninvasively [60]. Many studies induced mental stress to investigate changes in this parameter, such as in [61] which observed a reduction in PAT during the Stroop word colour test, and [20] which reported the same behaviour during the Trier Social Stress Test (TSST).
3. **Respiratory parameters (Resp)** are also broadly studied in experimental research and used in interventions, such as biofeedback, addressed to reduce psychological symptoms present in anxiety and other mental disorders [62-63]. The respiratory rate (RR) is expected to increase during stress exposure; however, it is easily affected by speech [21]. The spectral peak (Pk) is studied as an indicator of respiration stability, therefore, the higher the peak, the more stable the respiration [64].
4. **Electrodermal activity (EDA)** is another common biosignal selected to the assess stress level in mental health disorders, more precisely it reflects changes in the electrical properties of the skin induced by the sweat glands, which are under the direct control of the sympathetic branch of the ANS [22]. This signal can be decomposed into a phasic, i.e., the fast-changing component in response to external stimuli, and a tonic component, i.e., the slow-varying baseline EDA which is an indicator of the individual’s psychophysiological state. Importantly, a parameter called EDASymp, which could be extracted from the EDA signal employing frequency spectral analysis, corresponds to sympathetic dynamics in non-exercise conditions and has also been reported as a reliable index to sympathetic activation in stress elicitation studies [57]. For instance, previous studies reported a significant increase in tonic component and EDASymp when subjects are submitted to the Stroop test or other mental stress tasks [59,65].
5. **Skin temperature (ST)** is also associated with changes in ANS, as the body temperature is controlled in response to stress. Acute stress provokes peripheral vasoconstriction, directing the blood into the core to protect vital organs, increasing core body temperature, and also decreasing distal temperature, thus the direction of temperature changes in a stress-inducing task depends on the site of measurement. For instance, on distal regions, like fingertips, previous studies revealed a significant decrease in temperature and, conversely, when sensors were placed on proximal sites, such as the neck, the temperature showed an increase [23,24]. In our study, we will investigate whether the changes in peripheral temperature occur in response to the Stroop test, taking into account possible differences in basal temperature between healthy and those diagnosed with mental health disorders.
